# Supplementary material for: The causal relationship between the human gut microbiota and pyogenic arthritis: a Mendelian randomization study
Source: Front Cell Infect Microbiol. 2024 Nov 26;14:1452480. doi: 10.3389/fcimb.2024.1452480 (PMC11629706; doi:10.3389/fcimb.2024.1452480)
Supplement: Supplementary file 1 [file DataSheet1.zip › mendelian test/ebi-a-GCST90027630.csv_forest.pdf]

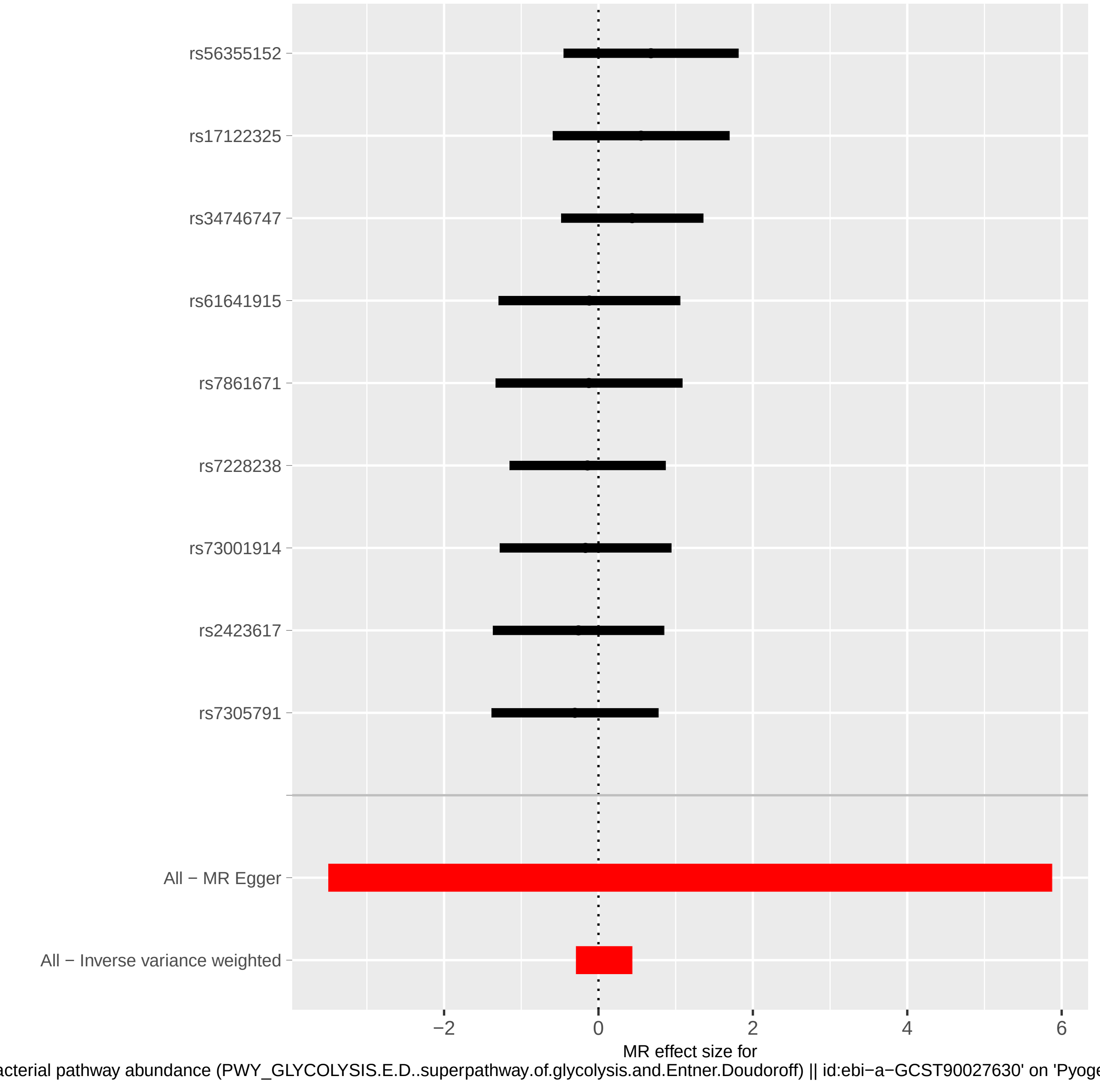
rs56355152
rs17122325
rs34746747
rs61641915
rs7861671
rs7228238
rs73001914
rs2423617
rs7305791
All − MR Egger
All − Inverse variance weighted
−2
0
2
4
6
MR effect size for
acterial pathway abundance (PWY_GLYCOLYSIS.E.D..superpathway.of.glycolysis.and.Entner.Doudoroff) || id:ebi−a−GCST90027630' on 'Pyoge
